# Outcomes of primary intestinal anastomosis versus stoma in necrotizing enterocolitis: A systematic review and meta-analysis

**DOI:** 10.1016/j.sipas.2025.100319

**Published:** 2025-11-10

**Authors:** Amani N. Alansari, Salma Mani, Marwa Messaoud, Tariq Altokhais

**Affiliations:** a Department of Pediatric Surgery, Hamad Medical Corporation, Doha, Qatar; b Pediatric Surgery Department, Fattouma Bourguiba University Hospital, Monastir, Tunisia; c University of Monastir, Faculty of Medicine of Monastir, Monastir, Tunisia; d Research Laboratory of Congenital Anomalies and Childhood Cancer LR12SP13, University of Monastir, Monastir, Tunisia; e Division of Pediatric Surgery, Department of Surgery, King Saud University Medical City, Riyadh, Saudi Arabia

**Keywords:** Necrotizing enterocolitis, NEC, Stoma, Primary anastomosis, Meta-analysis

## Abstract

**Background:**

Approximately one-quarter of necrotizing enterocolitis (NEC) cases require surgical resection due to bowel perforation, necrosis, or failure to respond to conservative management. In such cases, the optimal method for restoring intestinal continuity remains debatable. Stoma is traditionally favored over primary anastomosis for its perceived safety, particularly in unstable infants, but it is associated with complications such as fluid and electrolyte imbalances, impaired growth, and the need for a second surgery. This study aims to systematically review and analyze the evidence comparing stoma versus primary anastomosis in neonates undergoing surgery for NEC.

**Methods:**

We searched PubMed, Web of Science (WOS), the Cochrane Library, and Scopus for studies comparing the outcomes of stoma versus primary anastomosis in neonates with NEC. The primary outcomes included overall postoperative complications, intestinal perforation, stricture, mortality, wound infection, time to full enteral nutrition, time to end parenteral nutrition, and length of hospital stay. The meta-analysis was conducted using Review Manager (RevMan) version 5.4.

**Results:**

Eighteen studies were included in the meta-analysis. Primary anastomosis was associated with lower mortality risk: (risk ratios (RR) = 0.61; 95 % confidence interval (CI): 0.42:0.88). No significant differences were observed between groups in overall complications, wound infection, duration of hospital stay, duration of parenteral nutrition, the need for a second operation (excluding stoma closure), strictures, and perforations.

**Conclusion:**

Primary anastomosis for neonates with NEC is associated with lower mortality and comparable complication rates compared to stoma formation in selected cases.

## 1 Introduction

Necrotizing enterocolitis (NEC) is a serious gastrointestinal disease in neonates, primarily affecting preterm infants, particularly those with very low birth weight. While many cases respond to medical management, approximately one-quarter require surgical intervention due to complications such as bowel perforation, necrosis, or failure to improve with conservative treatment. Severe cases can be life-threatening, often requiring urgent surgical care [1,2]. Although it can also develop in full-term neonates, prematurity remains the most significant risk factor due to impaired mucosal integrity, immature gut immunity, and altered microbial colonization [3]. It was estimated that 5–10 % of infants born weighing less than 1500 g are affected [4]. Etiology involves multiple factors, including intestinal ischemia, dysbiosis, and formula feeding [5,6]. The prognosis varies depending on disease severity and extent of bowel involvement, with high morbidity and mortality rates despite advances in neonatal care. The reported overall mortality rates ranged from 10 % to 50 % [1,7]. Management of NEC extends from medical therapy in the early stages to surgical interventions in severe, complicated cases [8,9]. The primary objectives of surgical intervention in NEC are to control sepsis and excise necrotic intestinal segments, with an emphasis on preserving maximal bowel length. After resection, the two main surgical strategies are the creation of a stoma or performing a primary anastomosis [10]. Traditionally, temporary stoma has been favored, especially in unstable infants or when there is a grossly inflamed and friable bowel [9]. Stoma offers more safety and a lower risk of anastomotic leak, allowing time for distal bowel resting before planned re-anastomosis. Nevertheless, the stoma is associated with some drawbacks, such as fluid and electrolyte losses, impaired weight gain, dehiscence, stricture, prolapse, skin complications, neurodevelopmental impairment, and the need for a second surgery for stoma closure [[11], [12], [13], [14]].

Previous reports have suggested primary anastomosis as a valid alternative, showing comparable and even superior results to stoma in selected neonates [15,16]. This approach avoids stoma-related complications and drawbacks, potentially offering advantages regarding recovery and shorter length of stay [16]. On the other hand, concerns regarding the increased risk of postoperative leakage have remained a barrier to its widespread use. Hence, an algorithm for careful patient selection is essential to optimize the choice between both approaches. The evidence comparing the two is still inconclusive, and current practices vary among centers and countries [17]. Given these uncertainties, this systematic review and meta-analysis pooled the current evidence comparing outcomes of stoma versus primary anastomosis of the neonate undergoing surgery for NEC, aiming toward more precise elucidation regarding optimal surgical management and better patient-specific decision-making.

## 2 Materials and methods

This systematic review and meta-analysis was carried out in accordance with the recommendations provided by the PRISMA (Preferred Reporting Items for Systematic Reviews and Meta-Analyses) guidelines [18].

### 2.1 Search strategy and study selection

A comprehensive systematic literature review was conducted using PubMed, the Cochrane Library, Web of Science (WOS), and Scopus, covering all records from their inception until April 1, 2025. No restrictions or filters were applied. The search included the following terms: necrotizing enterocolitis, NEC, stoma, ileostomy, colostomy, enterostomy, and anastomosis. Details of the search strategy and results are provided in **Table S1**. Three independent authors assessed the records for relevance. Articles deemed relevant based on title and abstract screening underwent a detailed full-text evaluation. Any disagreements were resolved through consensus with a fourth author.

### 2.2 Eligibility criteria

We included comparative studies assessing the outcomes of primary anastomosis versus stoma formation in neonates diagnosed with NEC who were indicated for surgery. Only studies published in English were considered, with no restrictions on publication date. We excluded case reports, case series without a comparison arm, reviews, editorials, and conference abstracts. Studies that did not specify NEC as the surgical indication were excluded.

### 2.3 Data extraction and quality assessment

After agreeing on the final list of eligible studies, two researchers independently extracted relevant data from each study. The extracted data, including study summaries, baseline characteristics, and outcomes, were recorded in Excel sheets. Extracted key variables included the year of publication, study design, study location, sample size, follow-up duration, and primary outcomes. Baseline data included gender distribution, mean gestational age, birth weight, age at surgery, weight at surgery, and age at the disease onset. Outcomes were extracted from each study arm as event counts out of the total for dichotomous outcomes or as means and standard deviations for continuous outcomes. The primary outcomes of interest included any complications, stricture formation, bowel perforation, need for a second operation (excluding stoma closure), duration of hospital stay (including both neonatal intensive care unit and general ward admissions), time to discontinue parenteral nutrition, time to achieve full enteral feeding, wound infection, and mortality. The quality of included studies was assessed using the Newcastle–Ottawa Scale (NOS) for observational studies, the Risk of Bias 2 (RoB-2) tool for randomized controlled trials, and the Joanna Briggs Institute (JBI) Critical Appraisal Checklist for case series [[19], [20], [21]].

### 2.4 Statistical analysis

Quantitative analysis was conducted using Review Manager (RevMan) version 5.4. Dichotomous outcomes were analyzed using risk ratios (RR), while continuous outcomes were assessed using mean differences (MD), each with 95 % confidence intervals (CI). Results were considered statistically significant if the p-value was less than 0.05. A random-effects model was used as the primary analytical approach, given the expected clinical and methodological heterogeneity across studies, including variations in study design, patient characteristics, and intervention criteria. This model accounts for both within- and between-study variability. Heterogeneity across studies was evaluated using the I² statistic, with thresholds interpreted as outlined in the Cochrane Handbook [22]. The risk of publication bias was assessed by visual inspection of funnel plots and statistically using Egger's test for funnel plot asymmetry.

## 3 Results

### 3.1 Search results

**Fig. 1:**
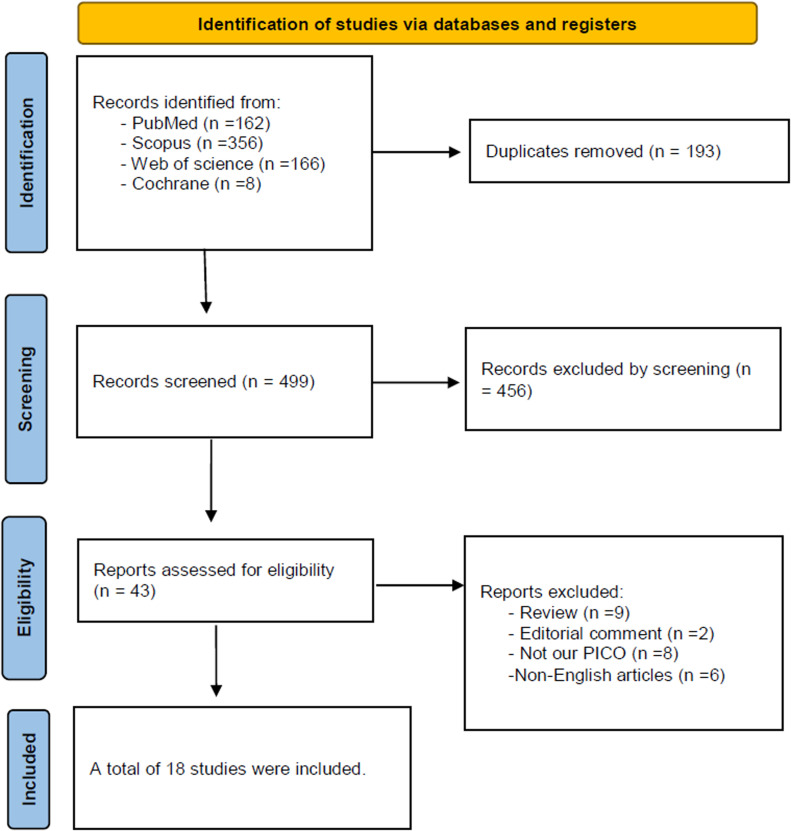
Prisma flow chart.

Our search identified a total of 692 records. After removing duplicates, 499 studies remained for the title and abstract screening, of which 43 were deemed relevant. Following a thorough full-text review, only 18 studies met the inclusion criteria and were incorporated into the meta-analysis [10,[14], [15], [16], [17],[23], [24], [25], [26], [27], [28], [29], [30], [31], [32], [33], [34], [35]]. The PRISMA flowchart is demonstrated in Fig. 1.

### 3.2 Study characteristics

The meta-analysis included a total of eighteen studies comprising one randomized controlled trial [27], one prospective cohort study [10], and sixteen retrospective cohort studies conducted in different parts of the world. The total of patients from all studies was 1361, with individual study populations ranging from 17 to 222 neonates. The primary outcomes assessed were diverse in each study, reflecting a broad spectrum of clinical interests. Twelve studies considered mortality as a primary outcome, while others focused on postoperative complications, duration of parenteral nutrition, and neurodevelopmental impairment. These details are demonstrated in **Table S2**. Across the included studies, gestational ages and birth weights varied widely, reflecting the heterogeneous neonatal populations. Most infants were born preterm, with the mean age ranging between 25 and 32 weeks. As shown in Table 1, the lowest reported gestational age was 25 weeks [27,30], while the highest reached up to 36 weeks [24]. Birth weights were similarly variable, with most patients being infants with very low birth weights. Mean birth weights ranged from approximately 715 g to over 2600 g The smallest average birth weights were observed in the Alzamrooni and Goldfarb studies [17,27], while higher birth weights, including those above 2000 g, were seen in studies with later gestational age groups [24]. These differences likely reflect variations in patient selection, surgical indication, and center-specific practices across the study periods and settings.

**Table 1:** Baseline characteristics of the included studies.

ID	Study Groups, (n)	Sex (male)	Gestational age (weeks)	Birth weight (g)	Age at surgery (days)	Weight at surgery (gm)	Age at the onset of disease (days)
Alzamrooni 2025	Primary anastomosis (*n* = 40)	19 (47.5)	25.0 ± 1.66	780 ± 148	5.0 ± 7.0	791 ± 140	NA
	Stoma (*n* = 92)	58 (63)	25.0 ± 4.34	715 ± 158	13.5 ± 9.6	782 ± 127	NA
Eaton 2024	Stoma (*n* = 38)	27 (71)	28.7 ± 4.7	1264.2 ± 867	9.5 ± 12.5	1392.6 ± 871	8 ± 12.5
	Primary anastomosis (*n* = 34)	22 (65)	29.2 ± 4.8	1318.8 ± 771.3	9.5 ± 23.75	1433.6 ± 727	7 ± 22.5
Goldfarb 2023	Primary anastomosis (*n* = 35)	NA	26.5 ± 3.0	809.67 ± 229.57	61.33 ± 49.47	NA	NA
	Stoma (*n* = 187)	NA	27.8 ± 4.5	996.33 ± 546.14	33 ± 31.38	NA	NA
Karila 2018	Stoma (*n* = 103)	93 (71)	27	870	10 ± 1.67	NA	NA
	Primary anastomosis (*n* = 28)						
Ramaswamy 2016	Panintestinal NEC with no intervention (*n* = 2)	13 (54.167)	35.04 ± 4.25	1810.5 ± 585	20.29 ± 19.75	NA	NA
	NEC; Stoma (*n* = 16)						
	NEC; Primary anastomosis (*n* = 6)						
Eltayeb 2010	Primary anastomosis (*n* = 12)	NA	NA	NA	NA	NA	NA
	Stoma (*n* = 11)	NA	NA	NA	NA	NA	NA
Ta 2010	Stoma (*n* = 14)	10 (71.43)	31.9 ± 4.29	1664 ± 707.57	NA	NA	13.25 ± 7.9
	Primary anastomosis (*n* = 5)	2 (40)	29.1 ± 1.88	1222.5 ± 340.68	NA	NA	13 ± 6.58
Singh 2006	Primary anastomosis (*n* = 37)	NA	29.5 ± 4.6	1287.5 ± 844.5	NA	NA	NA
	Stoma (*n* = 28)	NA	28.9 ± 4.5	1121.4 ± 732.9	NA	NA	NA
Hall 2005	Primary anastomosis (*n* = 12)	10 (83.33)	25.25 ± 1.45	780 ± 121.79	NA	805 ± 104.92	14.25 ± 10.73
	Stoma (*n* = 14)	7 (50)	25.25 ± 1.45	730 ± 128.19	NA	775 ± 110.04	23.25 ± 16.75
Hofman 2004	Primary anastomosis (*n* = 34)	NA	30 ± 3	1194 ± 346	17 ± 12.04	NA	16 ± 12
	Enterostomy (*n* = 29)	NA	32 ± 4	1577 ± 781	8 ± 5.1	NA	7 ± 5
Fasoli 1999	Isolated NEC; Primary anastomosis (*n* = 18)	NA	31.25 ± 3.75	1698.75 ± 673.75	NA	NA	15.5 ± 10.5
	Isolated NEC; Stoma (*n* = 7)	NA	32 ± 4.08	2060 ± 935.59	NA	NA	7.75 ± 5.16
	Multifocal NEC; Primary anastomosis (*n* = 26)	NA	31 ± 3.5	1400 ± 707.5	NA	NA	7.5 ± 25.75
	Multifocal NEC; Stoma (*n* = 20)	NA	31 ± 4	1839 ± 911	NA	NA	15.25 ± 10.25
Ade-ajayi 1996	Primary anastomosis (*n* = 18)	12 (66.67)	31 ± 3.5	1494 ± 721	NA	NA	NA
	Stoma (*n* = 8)	6 (75)	28 ± 1.5	879 ± 170	NA	NA	NA
Parigi 1994	Stoma (*n* = 17)	NA	31 ± 3.5	1621 ± 815	13.5 ± 8.8	NA	11.7 ± 8.3
	Primary anastomosis (*n* = 6)	NA	32 ± 3.5	1457 ± 230		NA	
Griffiths 1989	Stoma (*n* = 13)	NA	32 ± 3.75	1808 ± 758	13 ± 14.53	NA	11 ± 11
	Primary Anastomosis (*n* = 29)	NA	32 ± 3.75	1871 ± 1136.14	11 ± 23.4	NA	9 ± 21.45
Cooper 1988	Primary anastomosis (*n* = 27)	NA	32.2 ± 3.5	1620 ± 720	NA	1680 ± 710	11.3 ± 12.6
	Stoma (*n* = 116)	NA			NA		
Sparnon 1987	Stoma (*n* = 7)	11 (64.7)	36 ± 2.45	2650 ± 465.3	13 ± 12.3	NA	10 ± 17.25
	Primary anastomosis (*n* = 10)		35 ± 2.67	2310 ± 506.67		NA	
Pokorny 1986	Primary anastomosis (*n* = 38)	NA	NA	NA	NA	NA	NA
	Stoma (*n* = 29)	NA	NA	NA	NA	NA	NA
Kiesewetter 1979	Primary anastomosis (*n* = 9)	6 (66.67)	NA	1352 ± 273	12 ± 3.75	NA	3.5 ± 1.625
	Stoma (*n* = 43)	NA	NA	NA	NA	NA	NA

Data are presented as numbers (percentages) or means ± standard deviations. Not reported (NR). * NICU stays.

### 3.3 Quality assessment

Most of the included studies were rated with good quality, demonstrating adequate selection, comparability, and outcome assessment. However, a few studies were rated as fair or poor in quality. The quality assessments for the observational studies and case series are summarized in **Tables S3&4**, respectively. The single randomized controlled trial was evaluated using the RoB-2 tool and was found to have some concerns of bias (*Figure S1*).

### 3.4 Meta-analysis

#### 3.4.1 Clinical outcomes

**Fig. 2:**
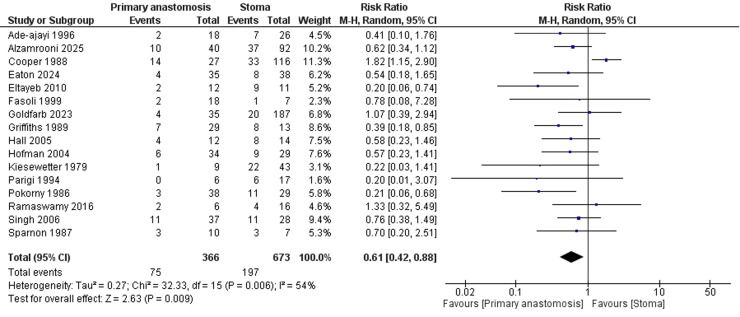
Forest plot comparing mortality rates between neonates undergoing primary anastomosis versus stoma formation for necrotizing enterocolitis.

A total of 16 studies reported mortality. The pooled analysis showed a significantly lower risk of *mortality* in the primary anastomosis group compared to the stoma group (RR = 0.61; 95 % CI: 0.42 to 0.88). Moderate heterogeneity among the included studies was detected (I² = 54 %) (Fig. 2). This heterogeneity was resolved after performing a leave-one-out sensitivity analysis, which showed that the exclusion of the study by Cooper et al. eliminated the observed heterogeneity (I² = 0 %) [29].

**Fig. 3:**
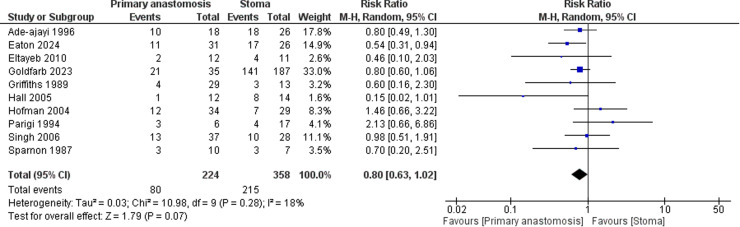
Forest plot comparing overall complications between neonates undergoing primary anastomosis versus stoma formation for necrotizing enterocolitis.

Primary anastomosis was associated with comparable *overall complication events* (RR = 0.80; 95 % CI: 0.63 to 1.02). There was non-significant heterogeneity among the included studies (I² = 18 %) (Fig. 3).

**Fig. 4:**
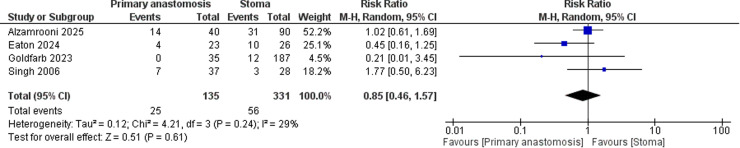
Forest plot comparing wound infection rates between neonates undergoing primary anastomosis versus stoma formation for necrotizing enterocolitis.

Four studies reported data on wound infection. The pooled analysis showed no statistically significant difference in the risk of *wound infection* between primary anastomosis and stoma formation (RR = 0.85; 95 % CI: 0.46 to 1.57). Heterogeneity was low (I² = 29 %) (Fig. 4).

The *duration of parenteral nutrition* (*Figure S2*) and *the time needed to achieve full enteral nutrition* (*Figure S3*) were comparable in both groups (MD = −1.84 days; 95 % CI: −4.15.1 to 0.47; I² = 0 %) and (MD = −16.81 days; 95 % CI: −43.27 to 6.64; I² = 96 %), respectively.

#### 3.4.2 Surgical outcomes

**Fig. 5:**
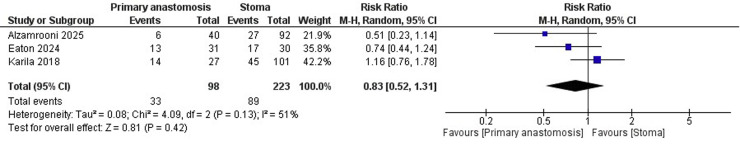
Forest plot comparing the need for second operation in neonates undergoing primary anastomosis versus stoma formation for necrotizing enterocolitis.

The need for a *second operation (excluding stoma closure)* was comparable in both groups (RR = 0.83; 95 % CI: 0.52 to 1.31). There was significant moderate heterogeneity among the included studies (I² = 51 %), which was resolved by excluding the study by Karila et al. [31] (Fig. 5).

**Fig. 6:**
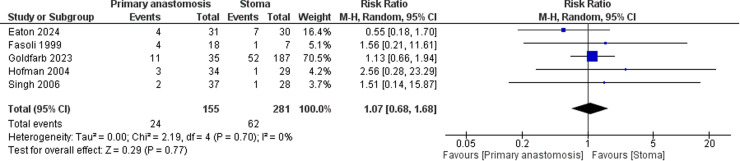
Forest plot comparing stricture rates between neonates undergoing primary anastomosis versus stoma formation for necrotizing enterocolitis.

**Fig. 7:**
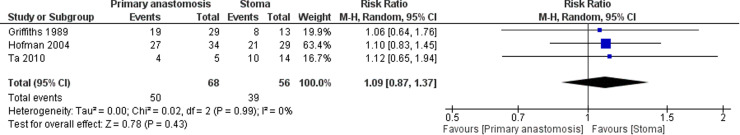
Forest plot comparing perforation rates between neonates undergoing primary anastomosis versus stoma formation for necrotizing enterocolitis.

There was no significant difference in the incidence of *strictures* (Fig. 6) or *perforations* (Fig. 7): (RR = 1.07; 95 % CI: 0.68 to 1.68) and (RR = 1.09; 95 % CI: 0.87 to 1.37), respectively. There was no significant heterogeneity among the included studies (I² = 0 %)

**Fig. 8:**
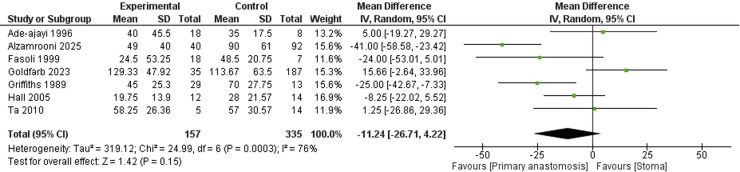
Forest plot comparing the length of hospital stay between neonates undergoing primary anastomosis versus stoma formation for necrotizing enterocolitis.

Similarly, *the length of hospital stay* did not differ markedly (MD = −11.24 days; 95 % CI: −26.71 to 4.22). Heterogeneity was considerable (I² = 76 %) (Fig. 8).

**Publication bias**

For all outcomes except mortality, the results of the funnel plots showed that the studies were symmetrically distributed around the pooled effect estimate, and Egger's test p-values were not significant, suggesting no evidence of publication bias or small-study effects for these outcomes. However, for the mortality outcome, Egger’s test yielded a significant p-value (*p* = 0.017), indicating probable publication bias even though the funnel plot did not show clear visual asymmetry (*Figures S4-S12*).

## 4 Discussion

Approximately one-quarter of necrotizing enterocolitis (NEC) cases are complicated and necessitate surgical intervention [2]. This systematic review and meta-analysis evaluated the outcomes of primary anastomosis versus enterostomy in neonates undergoing intestinal resection for NEC. We observed comparable outcomes in terms of complications such as perforation, wound infection, strictures, and the need for a second operation. Moreover, although not statistically significant in all studies, there was a consistent trend toward earlier establishment of enteral feeding, reduced parenteral nutrition duration, and shorter hospital stays in the anastomosis group.

The only meta-analysis in this topic was published in 2017 by Haricharan et al., reporting similar findings of a lower risk of mortality with primary anastomosis compared to stoma formation [36]. However, all studies included in that meta-analysis, and most of the studies in our own, were retrospective, introducing several limitations. For example, the higher complications and mortality observed in the stoma group may reflect more disease severity in those patients rather than the impact of the procedure itself. Our meta-analysis included more recent studies and the latest randomized controlled trial to deliver an updated and thorough comparison.

Our pooled analysis reinforces the conclusions of the recent trial by Eaton et al., which enrolled eighty infants with suspected NEC requiring laparotomy [26]. During surgery, if both procedures were considered feasible by the operating surgeon, infants were randomized to receive either primary anastomosis or stoma. The study found that infants undergoing primary anastomosis achieved enteral autonomy significantly earlier than those in stoma group. However, both groups showed comparable findings in terms of complications requiring unplanned reoperations. Notably, the trial revealed no significant difference in mortality between the two groups, suggesting that the survival advantage previously attributed to primary anastomosis was a biased finding in the retrospective studies. Therefore, in settings where both surgical options are viable, the choice of primary anastomosis should not be based on presumed mortality benefit but rather on its advantages in promoting faster recovery, avoiding stoma-related complications, and eliminating the need for a second operation for stoma closure [26]. The largest retrospective study from the United States, conducted by Goldfarb et al., highlighted the low frequency of primary anastomosis in clinical practice [17]. The study included 222 infants with surgical NEC, of whom only 15.8 % underwent primary anastomosis. Outcomes such as wound infection rates, duration of parenteral nutrition, and length of hospital stay were comparable between the primary anastomosis and stoma groups. The inclination toward stoma formation, rather than primary anastomosis, likely reflects a cautious surgical approach intended to mitigate postoperative risks, especially in unstable or low-birth-weight neonates. The choice of stoma creation, in addition to the factors previously mentioned, is often influenced by concerns over the ongoing NEC process, even after surgical intervention. This has led many clinicians to opt for stoma formation over primary anastomosis as a safer option in cases where the severity of the disease is uncertain or the risk of disease progression is high [15,17]. Another important consideration is the management of refeeding. Refeeding through a distal stoma offers controlled nutritional support while minimizing complications from prolonged total parenteral nutrition (TPN), such as liver disease. This approach is particularly beneficial for neonates at high risk of feeding intolerance, providing a more manageable alternative and strengthening the rationale for stoma creation in specific cases [37]. Nevertheless, these concerns were thought to be overestimated in many cases where primary anastomosis could be a safe and beneficial option. This emphasizes the importance of establishing selection criteria to optimize surgical management.

Building a reliable evidence-based decision-making algorithm requires further research on specific patient subgroups, such as different age groups, disease severity, and complication profiles. To help address this gap, Alzamrooni and colleagues conducted a study targeting extremely low birth weight preterm infants with perforated NEC [27]. They included infants (birthweight <1000 g) who had laparotomy for NEC managed with primary anastomosis or stoma formation. Primary anastomosis was associated with shorter hospital stays and more rapid achievement of enteral autonomy. Mortality rates were similar between the two groups. Interestingly, among micro-preemies (birth weight <750 g at surgery), outcomes did not differ significantly between primary anastomosis and stoma formation [27]. This highlights that age or extreme prematurity may not necessarily preclude the use of primary anastomosis. One of the other studied factors is the extent of disease. Previously, Fasoli et al. found that performing a primary anastomosis after bowel resection is a suitable therapeutic option for managing both localized and multifocal NEC, indicating its potential applicability even in cases with multiple affected intestinal segments [25]. In line with that, Eaton et al. advocated for primary anastomosis as the preferred approach, provided there is no disease distal to the resection site [26]. They reported that this procedure can be safely implemented across all weight categories, including infants requiring mechanical ventilation or inotropic support, as well as those with localized or multifocal intestinal involvement [[25], [26]].

Despite the consistent findings, several limitations should be acknowledged. First, the predominance of retrospective and non-randomized studies introduces substantial selection bias, particularly related to surgical decision-making. In most cohorts, the choice between primary anastomosis and stoma formation was not random but guided by the infant’s clinical status, bowel viability, and intraoperative findings. Consequently, more critically ill or unstable neonates were more likely to undergo stoma formation, which inherently predisposes this group to higher complication and mortality rates, independent of the surgical technique itself. Second, with only one randomized controlled trial available, the overall certainty of evidence remains low to moderate. Moreover, unmeasured confounders such as gestational age, birth weight, sepsis severity, and extent of bowel involvement may have further influenced outcomes but were inconsistently reported across studies. Finally, long-term parameters such as growth and neurodevelopmental outcomes were infrequently assessed, limiting conclusions regarding the sustained impact of the surgical approach.

## 5 Conclusion

Primary anastomosis for neonates with NEC is associated with lower mortality and comparable complication rates compared to stoma formation in selected cases. There is a clear need for large-scale, multicenter randomized trials to confirm these findings and guide evidence-based surgical decision-making. Future research should also evaluate long-term outcomes, caregiver burden, and healthcare costs associated with each approach.

## Funding

This research did not receive any specific grant from funding agencies in the public, commercial, or not-for-profit sectors.

## Availability of data and materials

The data that support the findings of this study are available upon reasonable request.

## CRediT authorship contribution statement

**Amani N. Alansari:** Writing – review & editing, Writing – original draft, Project administration, Methodology, Investigation, Formal analysis, Data curation, Conceptualization. **Salma Mani:** Writing – review & editing, Writing – original draft, Visualization, Validation, Investigation, Data curation. **Marwa Messaoud:** Writing – review & editing, Validation, Investigation, Formal analysis, Data curation. **Tariq Altokhais:** Writing – review & editing, Visualization, Validation, Methodology, Conceptualization.

## Declaration of competing interest

The authors declare that they have no known competing financial interests or personal relationships that could have appeared to influence the work reported in this paper.

## Appendix Supplementary materials

Image, application 1
