# Supplementary material for: Outcomes of primary intestinal anastomosis versus stoma in necrotizing enterocolitis: A systematic review and meta-analysis
Source: Surg Pract Sci. 2025 Nov 10;23:100319. doi: 10.1016/j.sipas.2025.100319 (PMC12670917; doi:10.1016/j.sipas.2025.100319)

**Supplementary Table 1.** Search strategy and search results.

| Database | Search strategy | Results |
| --- | --- | --- |
| PubMed | ("necrotizing enterocolitis" OR "necrotising enterocolitis" OR NEC) AND (stoma* OR ostom* OR ileostom* OR colostom* OR enterostom*) AND (anastomo* OR shunt OR bypass OR inosculat* OR conjunction OR junction OR fusion) | 162 |
| Scopus | TITLE-ABS-KEY ( ( "necrotizing enterocolitis" OR "necrotising enterocolitis" OR nec ) AND ( stoma* OR ostom* OR ileostom* OR colostom* OR enterostom* ) AND ( anastomo* OR shunt OR bypass OR inosculat* OR conjunction OR junction OR fusion ) ) | 356 |
| WOS | ALL=((“necrotizing enterocolitis” OR “necrotising enterocolitis” OR NEC) AND (stoma* OR ostom* OR ileostom* OR colostom* OR enterostom*) AND (anastomo* OR shunt OR bypass OR inosculat* OR conjunction OR junction OR fusion)) | 166 |
| Cochrane | ((“necrotizing enterocolitis” OR “necrotising enterocolitis” OR NEC) AND (stoma* OR ostom* OR ileostom* OR colostom* OR enterostom*) AND (anastomo* OR shunt OR bypass OR inosculat* OR conjunction OR junction OR fusion)) | 8 |

**Supplementary Table 2**. Summary of the included studies.

| **ID** | **Study design** | **Country** | **Total number of patients** | **Follow-up duration (months)** | **Primary Outcome** |
| --- | --- | --- | --- | --- | --- |
| Alzamrooni 2025 | Retrospective cohort | Canada | 132 | 12 | Mortality |
| Eaton 2024 | Randomized controlled trial | multicenter worldwide | 80 | 3, 12 | The duration of parenteral nutrition |
| Goldfarb 2023 | Retrospective cohort | United States | 222 | > 3.3 | Mortality |
| Karila 2018 | Retrospective cohort | Finland | 142 | 3 | Mortality |
| Ramaswamy 2016 | Retrospective cohort | Saudi Arabia | 24 | 3 | Mortality |
| Eltayeb 2010 | Prospective cohort | Egypt | 35 | 2-6 | Risk factors |
| Ta 2010 | Retrospective cohort | The Netherlands | 21 | > 72 | Intelligence score |
| Singh 2006 | Retrospective cohort | United Kingdom | 68 | NR | Postoperative complications |
| Hall 2005 | Retrospective case series | England | 51 | 4.7-48.4 (mean: 34.2) | Short- and long-term survival |
| Hofman 2004 | Retrospective cohort | The Netherlands | 63 | 6 - 115 | Postoperative complications |
| Fasoli 1999 | Retrospective cohort | England | 83 | 1 - 117 (mean: 42) | Survival rate |
| Ade-ajayi 1996 | Retrospective cohort | England | 26 | 24 | Mortality |
| Parigi 1994 | Retrospective cohort | Italy | 28 | 48-156 | Mortality |
| Griffiths 1989 | Retrospective cohort | Western Australia | 50 | 6 - 148 (mean: 60) | Mortality |
| Cooper 1988 | Retrospective cohort | USA | 143 | NR* | Mortality |
| Sparnon 1987 | Retrospective cohort | England | 17 | NR | Mortality |
| Pokorny 1986 | Retrospective cohort | USA | 78 | 13 | Mortality |
| Kiesewetter 1979 | Retrospective cohort | Pennsylvania | 98 | 36 | Mortality |

NR: Not reported.

**Supplementary Table 3**. Quality assessment of observational studies by NOS.

| Study ID | Selection | | | | Comparability | Outcome | | | Quality score |
| --- | --- | --- | --- | --- | --- | --- | --- | --- | --- |
|  | D1 | D2 | D3 | D4 |  | D5 | D6 | D7 |  |
| Alzamrooni 2025 | * | * | * | * | * | * |  | * | Good |
| Goldfarb 2023 | * | * | * | * | ** | * | * | * | Good |
| Karila 2018 | * | * | * |  | * | * | * | * | Good |
| Ramaswamy 2016 | * | * | * | * | * | * | * |  | Good |
| Eltayeb 2010 | * |  | * | * |  | * |  | * | Poor |
| Ta 2010 |  | * | * | * | ** | * | * | * | Good |
| Singh 2006 | * | * | * | * | ** | * | * |  | Good |
| Hofman 2004 | * | * | * | * | * | * | * | * | Good |
| Fasoli 1999 | * | * | * | * | ** |  | * | * | Good |
| Cooper 1988 | * | * | * | * |  | * | * | * | Poor |
| Ade-ajayi 1996 | * | * | * | * | * | * | * | * | Good |
| Parigi 1994 | * | * | * | * | * | * | * | * | Good |
| Griffiths 1989 | * | * | * | * | * | * | * | * | Good |
| Sparnon 1987 |  |  | * | * | * | * |  | * | Fair |
| Pokorny 1986 | * | * | * | * |  | * | * | * | Poor |
| Kiesewetter 1979 | * |  | * | * | * | * | * | * | Good |

D1: Is the case definition adequate/Representative of the exposed cohort?

D2: Representative of the cases/Selection of the non-exposed cohort.

D3: Selection of Controls/Ascertainment of exposure.

D4: Definition of Controls/ Demonstration that outcome of interest was not present at start of study.

D5: Ascertainment of exposure/ Assessment of outcome.

D6: Same method of ascertainment for cases and controls/ Was follow-up long enough for outcomes to occur.

D7: Non-Response rate/ Adequacy of follow-up of cohorts.

**Supplementary Table 4**. Risk of bias of the case series study by JBI.

| **Study ID** | **D1** | **D2** | **D3** | **D4** | **D5** | **D6** | **D7** | **D8** | **D9** | **D10** | **Overall Quality Assessment** |
| --- | --- | --- | --- | --- | --- | --- | --- | --- | --- | --- | --- |
| **Hall 2005** | yes | yes | yes | yes | yes | yes | yes | yes | yes | Not applicable | Include |

D1 - Were there clear criteria for inclusion in the case series?

D2 - Was the condition measured in a standard, reliable way for all participants included in the case series?

D3 - Were valid methods used for identification of the condition for all participants included in the case series?

D4 - Did the case series have consecutive inclusion of participants?

D5 - Did the case series have complete inclusion of participants?

D6 - Was there clear reporting of the demographics of the participants in the study?

D7 - Was there clear reporting of clinical information of the participants?

D8 - Were the outcomes or follow-up results of cases clearly reported?

D9 - Was there clear reporting of the presenting site(s)/clinic(s) demographic information?

D10 - Was statistical analysis appropriate?

**Supplementary Figure 1.** Risk of bias of the RCT study by ROB-2.


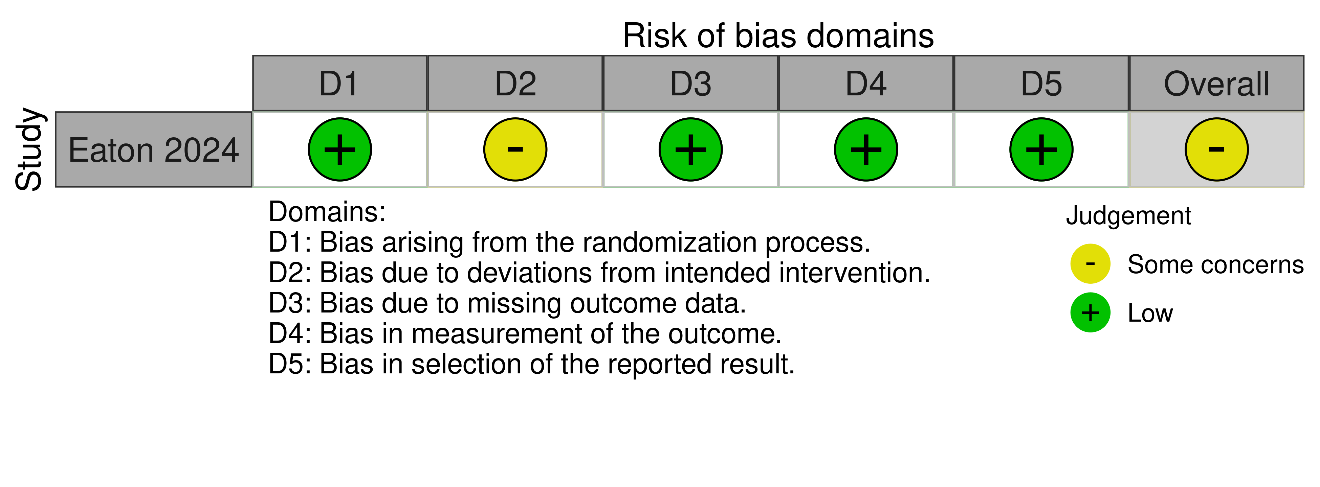


**Supplementary Figure 2**. Forest plot comparing the duration of parenteral nutrition between neonates undergoing primary anastomosis versus stoma formation for necrotizing enterocolitis.


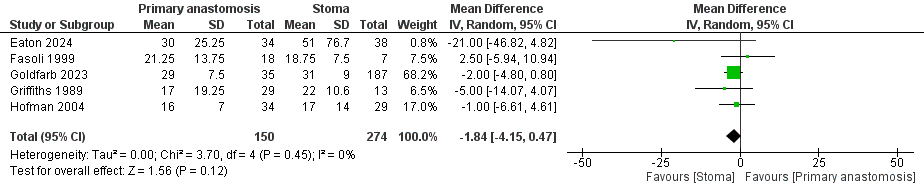


**Supplementary Figure 3**. Forest plot comparing the time to full enteral nutrition between neonates undergoing primary anastomosis versus stoma formation for necrotizing enterocolitis.


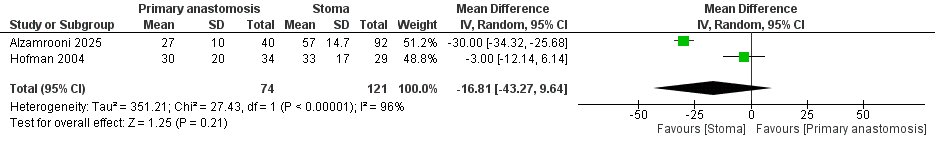


**Figure S4.** Funnel plot of the mortality rates outcome.


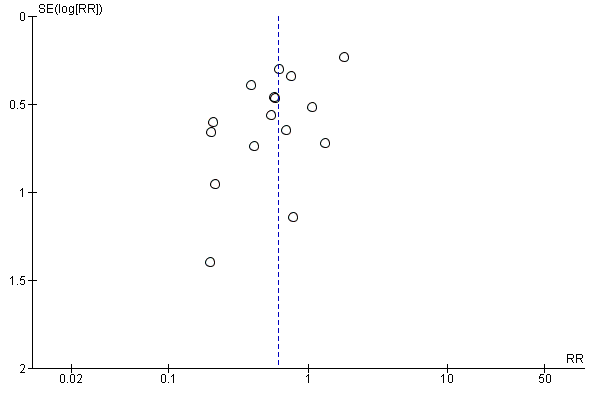


**Figure S5.** Funnel plot overall complications outcome.


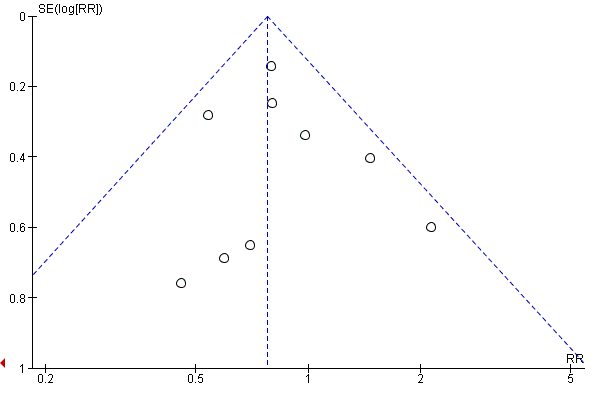


**Figure S6.** Funnel plot of wound infection rates outcome.


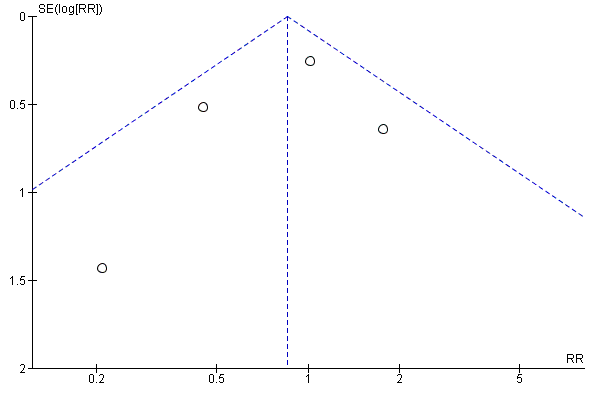


**Figure S7.** Funnel plot of the duration of parenteral nutrition outcome.


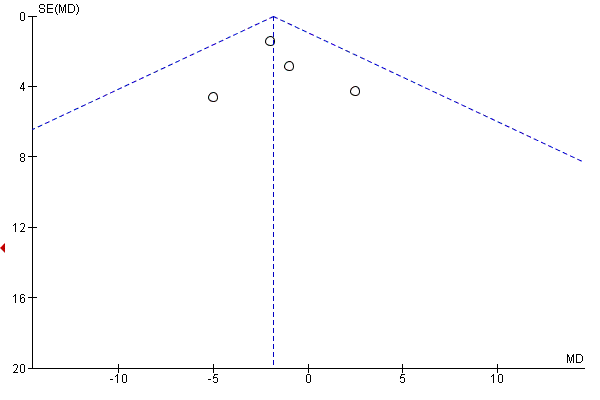


**Figure S8.** Funnel plot of the time to full enteral nutrition outcome.

**
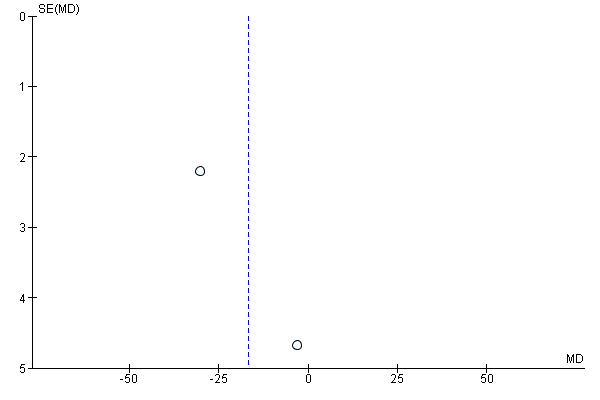
**

**Figure S9.** Funnel plot of the need for the second operation outcome.


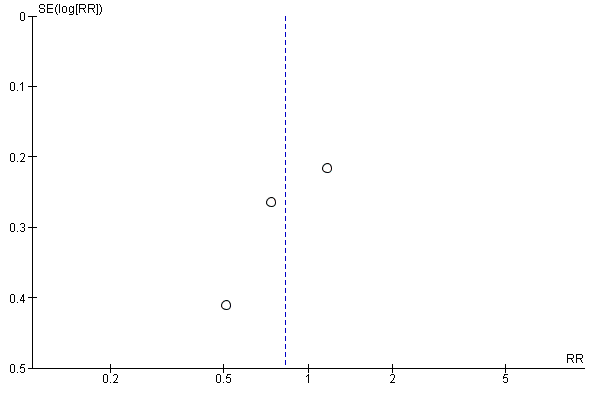


**Figure S10.** Funnel plot of the stricture rates outcome.


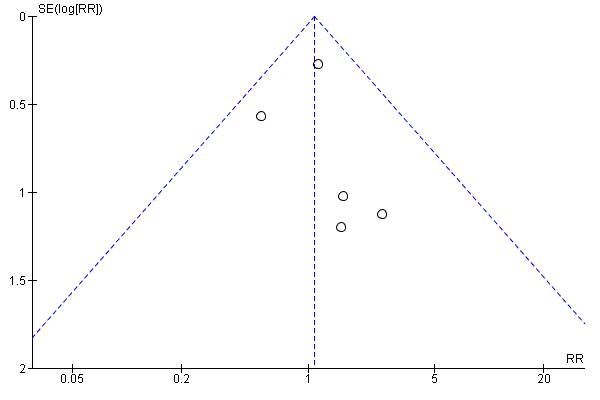


**Figure S11.** Funnel plot of the perforation rate outcome.


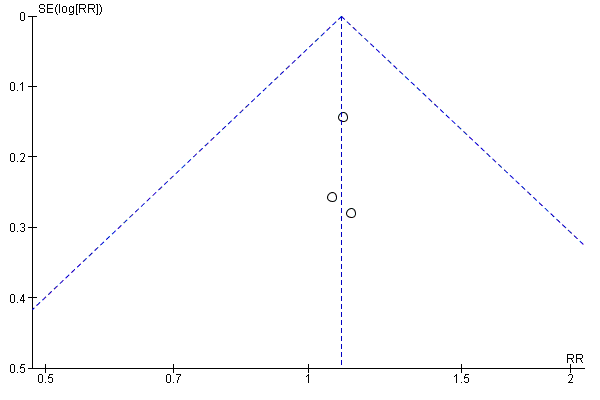


**Figure S12.** Funnel plot of the length of hospital stay outcome.


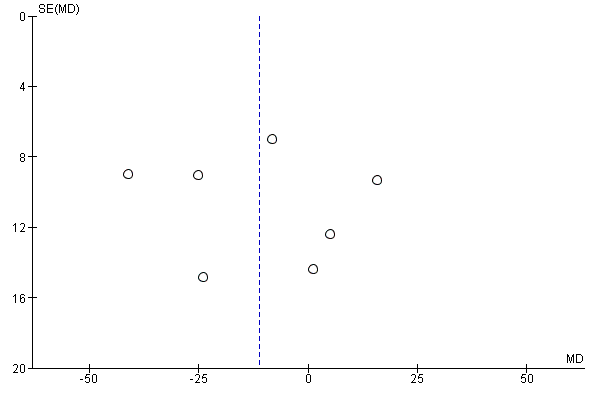

Supplement: Supplementary file 1 [file mmc1.docx]
